# Caregiving and Shared Decision Making in Breast and Prostate Cancer Patients: A Systematic Review

**DOI:** 10.3390/curroncol30010061

**Published:** 2023-01-06

**Authors:** Clizia Cincidda, Silvia Francesca Maria Pizzoli, Giulia Ongaro, Serena Oliveri, Gabriella Pravettoni

**Affiliations:** 1 Applied Research Division for Cognitive and Psychological Science, IEO, European Institute of Oncology IRCCS, 20141 Milano, Italy; 2 Department of Oncology and Hemato-Oncology, University of Milan, 20122 Milano, Italy

**Keywords:** caregivers, prostate cancer, breast cancer, decision-making

## Abstract

Background: A cancer diagnosis can impact patients’ and caregivers’ lives, posing different challenging situations. In particular, breast cancer and prostate cancer are two types of cancer involving families and especially spouses in challenges linked with the diagnosis and treatment process. Caregivers are usually involved in the treatment decision-making (TDM) process concerning patients’ clinical pathway, cancer treatment, and ongoing therapies. To date, no contributions provide an exhaustive overview of the role of caregivers in cancer care and their involvement in the TDM process related to the therapies. Methods: We performed a systematic review of caregiver and patients experiences and perceptions of caregiver involvement in cancer TDM. Articles were searched on Public/Publisher MEDLINE (PubMed), Excerpta Medica Database (Embase), Medical Literature Analysis and Retrieval System Online (Medline), and American Psychological Association APA PsycINFO. Results: 17 studies were included, 10 on prostate cancer and 7 on breast cancer. According to the reviewed studies, patients and caregivers experienced the cancer diagnosis with a sense of unity. Most patients preferred to have an active or collaborative role with caregivers in TDM, feeling it was important to consult or share the decision made with their caregivers. Caregivers preferred to collaborate with patients or let patients decide by themselves after considering their opinions. Caregiver involvement could have a positive influence on the patient’s medical decisions, even if cancer diagnosis and treatments overwhelmed patients and caregivers. Conclusions: These findings highlight the importance of using a perspective that focuses on the relationship between a patient and caregivers when they receive a cancer diagnosis and have to make a treatment decision. Targeting caregiver–patient dyads, rather than individuals, is important since a supported relationship could have a protective effect on psychological distress, quality of life (QOL), and relationship satisfaction. Moreover, dyads may benefit from interventions that focus on the needs of both the patient and caregiver.

## 1. Introduction

A cancer diagnosis and its subsequent treatments may psychologically and practically affect patients’ and caregivers’ lives, posing different challenging situations [1]. Quite often, caregivers are the spouse/partner or the adult child of the patients; in rare cases, even friends can play this role. Patients and their family members face the dramatic experience of cancer together by going through different stages of adjustment to cancer disease [2]. In particular, breast cancer, which predominantly affects women, and prostate cancer, which affects men, are two types of cancer involving families and especially spouses in challenges linked with diagnosis and the treatment process [3,4,5]. When a patient receives the bad news of a breast or prostate cancer diagnosis, a multitude of emotional reactions, including anger, fear, and a sense of guilt, are frequent and can take the form of anxious symptoms (agitation, concentration difficulties) and/or depressive symptoms (apathy, low mood tone) [6]. Specifically, breast cancer patients may have concerns about changes in body image, sexuality, or attractiveness, difficulties, and uncertainty in planning the future, and physical toxicities that result from adjuvant therapy but also changes to the social role or adjustment in family roles [7,8]. Breast cancer is also considered a serious blow to femininity [9,10,11]. In prostate cancer patients, based on the different stages of disease progression, concerns may emerge about sexual impotence (e.g., erectile dysfunction, reduced libido) and reproductive spheres or problems related to hormonal function or incontinence [4]. These aspects can have further negative psychological implications, causing anxiety, depression, fatigue, stress, pain, and fear of cancer recurrence [12,13,14,15]. The same psychological reactions can sometimes affect patients’ partners [5,16,17,18]. Indeed, both breast- and prostate-cancer-related symptoms and side effects due to the treatment can primarily impact the intimacy of the dyads. Subsequently, they might extend their negative effects to the wider social network of the cancer patients, which includes family members, friends, and anyone who performs the role of caregiver [5,19].

Along the cancer trajectory, care options are discussed, or decisions are made. The process by which people decide is called the decision-making (DM) process. The DM process is a reasoning process based on assumptions of values, preferences, and beliefs of the decision-maker (in this case the patient), producing a final choice [20]. Specifically, breast cancer patients generally undergo surgery and/or other therapies including chemotherapy, hormone therapy, and radiation therapy. In the case of early breast cancer, decisions may concern the type of surgery (e.g., mastectomy vs. lumpectomy), reconstructive surgery, and participation in clinical trials [21]. Regarding prostate cancer, patients are faced with the difficult decision of active surveillance, radiotherapy, or surgery [22]. In both cases, the best treatment for each patient depends on both medical factors and personal assessment of the risks and benefits of each choice. Over the past decades, there has been a shift from a paternalistic approach (the patient is a passive receiver of treatment) to a patient-centered paradigm (the patient is an active participant) that allows patients to be actively involved in the treatment decision-making (TDM), and more in general, along their clinical pathway [23,24]. This way, within the concept of patient-centered care emerged the concept of a shared decision-making process (SDM) that considers patients’ individual preferences, needs, and values, which in turn, guides all clinical decisions [25,26]. However, quite often, in the oncological field, patients are accompanied by their caregivers during the visits to support the patient in the interaction and communication with the oncologists (to help understand information on the disease and its consequences, prescribed therapies and expected outcomes, side effects, and the best way to control them) and provide emotional closeness. This way, caregivers may be involved in the TDM process, stating their opinions, preferences, and beliefs on the treatment decisions [27,28,29]. Patients are defined by their relationship and depend on “significant others” to make decisions, and in this case, they may take into consideration the caregiver standpoint [12,30,31,32]. The decision-making process concerning treatment and patient care is replete with uncertainty and anxiety both for the patients and the caregivers [33]. Caregiver involvement in the oncological examinations was associated with increased patient satisfaction with care, understanding of cancer-related information, treatment adherence, and physical and mental health [34,35,36,37]. This way, caregivers may influence patient decisions regarding medical treatments, bringing with them a range of emotional reactions, interpersonal dynamics, and expectations [28,32,37,38,39,40,41]. For that reason, clinicians are moving towards family-centered models of care and the need to develop a new line of research on dyadic decision-making in the oncological domain, considering the relationship between patients and caregivers [42,43]. Until now, there was no gold standard related to caregiver involvement. However, an alignment among patient–caregiver preferences for the extent of caregiver involvement in the decision-making process may enhance the process of care for all the parties [36,44].

To date, few contributions provide an exhaustive overview of the role of caregivers in cancer care, but none of them comprehensively address newly diagnosed cancer patient and caregiver involvement in the TDM process related to therapies. Already published systematic reviews concern caregiver involvement and support in palliative care or for elder patients, who need caregiver practical assistance in care and treatment, or concern the effectiveness of psychological intervention on the triadic relationship between patients, caregivers, and physicians aimed at enhancing the triadic communication [38,45,46,47,48,49,50]. In this literature review, we focused on newly diagnosed breast and prostate cancer for a pragmatic reason, being the two most common diseases in women and men, respectively, and on the TDM process by considering the fact that some decisions related to cancer therapies can also involve the sphere of intimacy and fertility and may have a direct impact on family caregivers, who play a fundamental role in the decision, insofar as the decision has an impact on them as well. Nevertheless, we did not select only contributions where partners as caregivers were involved since we wished to explore/assess whether there are also differences in dyadic dynamics and level of compliance/agreement due to the type of relationship. Our contributions can help to identify future directions for research concerning caregiver involvement in cancer care and hints for psychological interventions to improve patient–caregiver dyadic interactions.

## 2. Methods

### 2.1. Search Strategy

**Figure 1:**
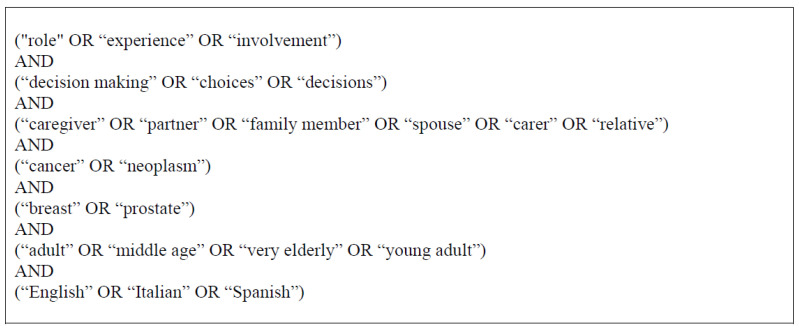
Database search terms.

The electronic search was performed through three major databases, including Public/Publisher MEDLINE (PubMed), Excerpta Medica Database (Embase), and American Psychological Association APA PsycINFO, between August to November 2020, and again in November 2022 to update the research, with no time limits. We followed PRISM guidelines to conduct our SR [51]. Original articles were considered in the English, Italian, or Spanish languages, with participants aged 18 years and up. The keywords searched in titles and abstracts included decision-making process, cancer treatment, prostate, and breast cancer, combined with terms such as caregiver involvement and patient–caregiver interaction. References in relevant systematic reviews were manually searched for additional contributions that met our inclusion criteria, although these systematic reviews were not focused on our same topic. Moreover, the reference lists of the records included were screened to identify additional relevant papers. The search was limited only to full-text articles. Due to the varied nature of keywords in this field, we developed a comprehensive list of search terms (see Figure 1 or Table A1). The present study was registered in the “Research Registry” in 2022 (ID: reviewregistry1498) and was conducted according to PRISMA guidelines.

### 2.2. Inclusion and Exclusion Criteria

Literature reviews, commentaries, editorials, book chapters, guidelines, and conference proceedings were not included in this review. An eligibility checklist summarizing the inclusion and exclusion criteria for article selection was developed (see Table 1). The studies included in this review met the following main criteria: (a) articles dealing with breast or prostate cancer patients and their caregivers, such as parents, spouses, sons, other loved ones, or close friends; (b) articles including any kind of psychological intervention promoting caregiver involvement in the breast and prostate cancer patient’s decision-making related to cancer treatment, as well as studies that did not include any psychological intervention (studies exploring/assessing the caregiver’s role in the breast and prostate cancer patient’s treatment decision-making). Regarding the research methods, we included quantitative, qualitative, and mixed methods empirical documents, assessing patient–caregiver decision-making related to cancer treatments. Specifically, qualitative research methods explain phenomena through in-depth interviews, semi-structured interviews, interviews, and focus groups; while quantitative research methods measure them through experiments, questionnaires, and surveys. The mixed methods research included both interviews or focus groups and surveys or questionnaires.

### 2.3. Screening Procedure

**Figure 2:**
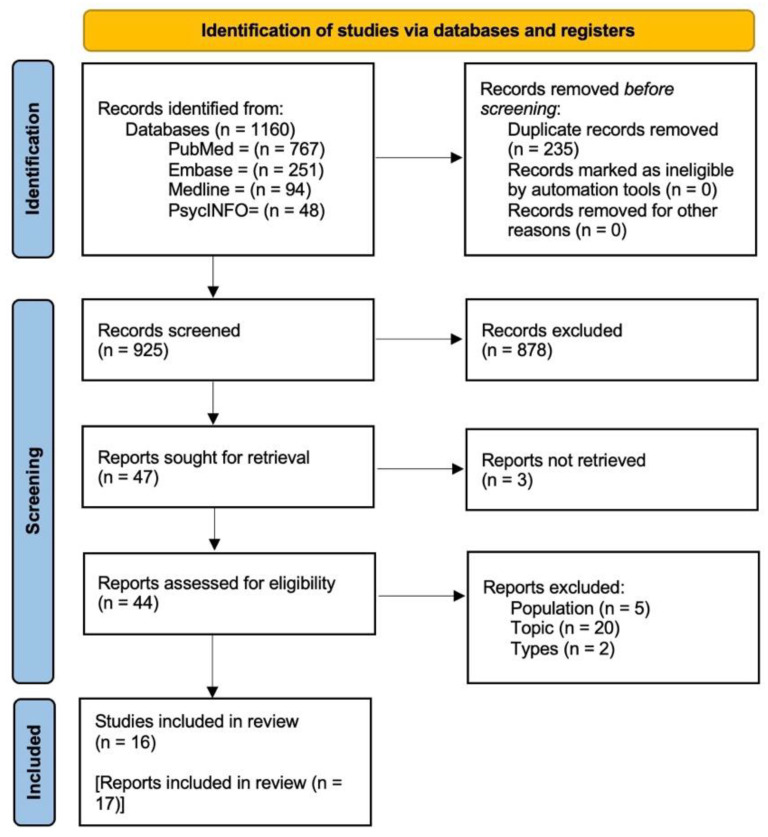
Preferred reporting items for systematic review and meta-analysis (PRISMA) statement flow diagram. [51].

Initially, the search strategy yielded 1160 records that were screened for irrelevant or duplicate records by the first author (CC). Prior to the selection process, all records were exported in EndNote X9 reference manager. The remaining records were assessed and selected by first screening the title and the abstract, followed by a full-text reading and selection according to the predefined inclusion and exclusion criteria. The entire selection process was performed by the first author (CC) and two co-authors (SFMP and GO) independently. In case of disagreement, the three authors tried to reach a consensus. Where needed, the fourth author (SO) was involved until a consensus was reached. If the full text of an article was not available, we emailed the first or corresponding author of that article to request a PDF copy. We also used the snowball technique and citation tracking to identify additional potentially relevant publications. We selected 17 reports focused on caregiver and patient experiences and perceptions of caregiver involvement in cancer TDM. Specifically, we included 16 studies, as two reports referred to the same study. The PRISMA flowchart of the selection procedures of the studies is reported in Figure 2 [51].

### 2.4. Data Extraction

Data were extracted from each of the included reports using a custom template: country of origin, study design, data collection technique, samples/population, measurement time-point, and significant findings. Initially, the first author (CC) and the second author (SFMP) independently read and reread all the included reports, underling the relevant parts for the purpose of the review. Subsequently, the two authors discussed together the relevant parts underlined to reach a consensus on what to extract from the reports. Then, data were extracted by the first author (CC) and cross-checked for accuracy by the second author (SFMP). Reports that were hard to decide were discussed between the researchers.

We identified 11 contributions using a qualitative method, 5 contributions using the quantitative method, and 1 contribution using the mixed method. Analyzing the significant findings from each report, we found another important aspect related to shared decision-making, so we identified two macro themes: patient and caregiver involvement in cancer decision-making, and the psychological impact and its reciprocal influence on cancer in patients and caregivers.

### 2.5. Risk of Bias Assessment

The quality of the eligible reports was assessed using the Downs and Black’s methodological quality scale [52], a 27-item scale that provide a score on the following methodological quality scale: the reporting bias, the external validity, the internal validity, the selection bias, and the power. An overall rating of “good”, “fair”, or “poor” was given for each report. All the studies were independently rated by CC and SFMP, and any disagreements were resolved through discussion with SO. Consistently with other previous reviews, the statistical power evaluation was simplified using only two scores (1 = the sample size required to detect the significant difference was calculated; 0 = the sample size was not calculated or was insufficient to reach adequate statistical power). For qualitative studies, we considered if an analysis plan with the required sample size or quantity data for the planned analysis was included in the paper. However, the contributions included in this SR received poor scores (5–12), which means a high risk of bias due to their research methodology. Indeed, the majority of these contributions used a qualitative method and none of the rest were a randomized control trial (RCT). Considering the lack of adequate literature on the caregiver role in treatment decision making, we consider it appropriate to report in this contribution the reports that have investigated the aforementioned aspects with qualitative and/or exploratory methodology.

## 3. Results

A total of 17 reports met the inclusion criteria, among which 5 used a quantitative research method [41,53,54,55,56], 11 used a qualitative research method [57,58,59,60,61,62,63,64,65,66,67], and 1 used a mixed-method research method [68]. The characteristics of these reports are summarized in Table 2 for the quantitative documents, in Table 3 for the qualitative documents, and in Table 4 for the mixed methods documents.

### 3.1. Characteristics of the Selected Reports

Five were carried out in the USA [41,57,62,64,67], three in Canada [53,54,60], five in Europe [58,59,61,63,65], one in Australia [66], one in Israel [55], one in China [56], and one in Oman [68].

Considering the quantitative reports, a total number of 1519 adult patient–caregiver dyads were analyzed. The majority of the dyads consisted of spouse–partner (54.51%; *n* = 828) or parent–son (18.24%; *n* = 277) with sons as caregivers, while the other dyads were composed of other family members (17.64%; *n* = 268, e.g., sister, cousin) or friends (8.03%; *n* = 122). The remaining 1.58% (*n* = 24) of caregivers did not provide any information about their relationship with patients. The majority of the patient sample was composed of women with breast cancer under medical treatment, such as surgery, at the initial phase of their illness (*n* = 1365; 89.86%). The time frame considered in the reports started from 3 to 12 months after a breast cancer diagnosis. Most women had surgery and were enrolled in the reports after breast reconstruction. Prostate cancer patients instead were enrolled at the time of diagnosis and had not yet decided on the treatment. Moreover, four reports were cross-sectional and only one study had a quasi-experimental design (pretest/post-test).

A total sample of 737 participants were interviewed in the qualitative reports, 400 (54.27%) were patients and 337 (45.73%) were caregivers. Caregivers were predominantly spouses/partners (*n* = 314, 93.18%), followed by other relatives (*n* = 16, 4.75%), among which 10 were sons; and only 3 caregivers were friends. Finally, nine caregivers (2.67%) did not specify their type of relationship with patients. Most patients had a prostate cancer diagnosis (*n* = 378; 94.5%). The methods employed in such reports were focus groups (1 study) and interviews (10 studies, 6 interviewing patients and caregivers separately, 5 interviewing the dyads together, and 1 mixed, interviewing them separately and together). One report used both methodologies (focus group and interview). Two of the thirteen reports had a longitudinal design, while the others were cross-sectional, except for one that was a pilot study.

One last report included in our review had a mixed methodology and enrolled 79 dyads in a time range of 24 months after the beginning of the treatment and included mostly dyads with spouses (39.2%), parents (39.2%), siblings or offspring (34.2%), and other relatives, such as nephews and stepsons (8.9%).

### 3.2. Shared Decision-Making between Patients and Caregivers

Reviewing quantitative studies, it was found that almost all the patients preferred to have an active or collaborative role with caregivers in the TDM process [53,54,55]. Indeed, patients felt it was important to make the final medical decision by themselves, even if only after consulting their caregiver or sharing the decisions with them [53,54,55]. Moreover, the majority of the patients reported that it was important for them to agree with their caregivers about the decision they made [55]. Finally, very few patients preferred to have a passive role, allowing their caregivers to decide [53,54,55]. Regarding the caregiver role in TDM, almost all the caregivers preferred to have a collaborative or passive role in the decision-making process [41,53,54]. However, most caregivers who took a passive role in the TDM process reported that patients decided after considering their opinion seriously [53]. Very few caregivers wanted to be active in deciding for the patients [54]. Another important aspect related to shared decision-making was the agreement between patients and caregivers. Only one study explored this topic and reported that patients and caregivers had similar ideas regarding both their attitudes toward their involvement in shared decision-making and their medical decisions [55]. Caregiver involvement in medical shared decision-making might have a positive influence on patients’ medical decisions, since greater caregiver involvement was associated with lower patient decision regret, even if the data were not statistically significant [56], and with an improvement of subjective decision quality and deliberation [41].

Regarding the results from qualitative studies, almost all the cancer patients stated that they preferred to discuss treatment options with their caregivers (e.g., partner, family members), giving importance to caregivers’ preferences, and that they felt supported by them in making treatment decisions [57,58,59,60,61,62,63,64,65,66,67]. Although patients involved caregivers in the decision-making process, feeling they were a unit/team [58,59,60,61,62,63,64,66,67], patients made the final treatment choice by themselves and the decision could be shared or not by caregivers [59,61,62,63,64,66]. Both patients and caregivers considered the decision-making process as personal and intimate, even if it was influenced by external factors, such as personalized care or psychological and emotional variables [59]. Moreover, it showed the importance of temporality in the decision-making process; some patients needed more time to decide than others [61,62]. Throughout the illness trajectory, caregivers provided emotional, instrumental and appraisal support, accompanying patients to medical appointments, remembering their screening appointments, helping them to manage daily life activities at home or to manage stressful events, encouraging and reassuring them [57,59,63,65,66,67]. In some cases, patients could perceive the caregivers’ support as inappropriate or they could feel a dissonance in their relationship; these enhanced concerns and caused conflicts [59,60].

Findings from the mixed methods study [68] reported that the majority of breast cancer patients discussed, finalized, and shared the medical decisions with caregivers (more than one family member among partners, siblings, or parents) when there was full agreement between patients and caregivers. Even caregivers reported to be involved in the TDM process and to have the role of supporting patients [68].

### 3.3. Psychological Impact of Cancer on Patient-Caregiver Dyads

Patients and caregivers consider a cancer diagnosis to be scary and severe, a true shock, trauma [60,62,64]. Frequently, patients and caregivers partially retreated from normal life at the beginning of their cancer journey full of concern regarding the future [60,64]. Then, patients and caregivers started to accept this new situation and tried to deal with it actively, searching for information as much as possible [57,58,60,62,64,65,66]. During the cancer trajectory, patients and caregivers lived with the illness experience as a shared challenge with a sense of being together and feeling it was important to discuss what scared them [59,60,63,64]. Patients identified a person in their family as a caregiver and received from them both emotional and instrumental support, attending and remembering medical appointments, giving reassurance, and/or hiding concern [57,63,64,65,66]. Moreover, caregivers needed to tend to their own emotional needs regarding the diagnosis to be strong for patients, and sometimes they found help in other family members or friends [57,64,65]. Indeed, patients and caregivers felt overwhelmed by the cancer diagnosis, the medical decision-making, treatments, and about their role [57,63,64]. However, when a treatment decision was made, some patients and caregivers tried to carry on as normal, while others continued to worry some of the time [60]. During the cancer trajectory, patients faced a lot of difficulties related not only to the diagnosis but also to the treatment and side-effects of treatments that were reported [61,63,65]. One of the most common concerns was related to sexuality or sexual intimacy, as well as who to tell about the cancer [60,61,63]. Due to the surgery and treatment, breast cancer patients had to face a change in their body, resulting in a loss of confidence in their nakedness [59,63]. However, male caregivers asserted that they did find their wives as attractive as before the cancer diagnosis [63]. Sometimes, patients and caregivers could disagree about whom to tell about the cancer or about seeking a second opinion or because patients perceived the support provided by caregivers as inappropriate, causing tension or conflict [59,60,66]. After the cancer diagnosis, patients underwent a great number of medical assessments to decide the best treatment, and during this period, the patient–caregiver dyad had to cope with the waiting time, and this was very anxiety-inducing, as much as when waiting for surgery [60,64]. Most of the patients and caregivers expressed a need for meeting other patients and caregivers in a similar situation to obtain psychosocial support during and after treatment [65]. Finally, the level of state-anxiety and depression that patients and caregivers experienced along the cancer trajectory decreased both in patients and caregivers over time. However, female caregiver state-anxiety levels were slightly higher than patient levels in both measurements. Finally, psychological distress did not affect the role that patients and caregivers wanted to play in medical decision-making vs. the role they actually assumed [53].

## 4. Discussion

Cancer can be considered a family disease [2], having an impact on both patients and caregivers, who face different challenging situations due to the cancer diagnosis [1,3,4,5]. Moreover, patients usually involve caregivers during the cancer consultation to be supported in the decision-making process, in the interaction and communication with the oncologists, and along the cancer trajectory [24,28,31,38]. With this contribution, we aimed to investigate breast and prostate cancer patient and caregiver involvement in the TDM processes related to the cancer care pathway, as well as their level of compliance. To date, no contributions provide an exhaustive overview of the role of caregivers in cancer care and their involvement in the decision-making process related to therapies.

Reviewing the studies on caregiver involvement in breast and prostate cancer patient decision-making, two macro themes emerged: the importance of patient and caregiver involvement in cancer decision-making and the psychological impact and reciprocal influence of cancer on patients and caregivers. Our review shows that most patients preferred to have an active or collaborative role with caregivers in TDM, and in both cases, patients felt the need to consult or share the decision made with their caregivers, even when caregivers did not agree with them [41,53,54,55,57,59,60,61,62,63,64,65,66,67,68]. Regarding the role of caregivers, they preferred to collaborate with patients or allow patients to decide by themselves after considering their opinions [41,53,54,55]. Only one study explored the agreement between patients and caregivers on their role in shared decision-making, reporting that patients and caregivers had similar views on both their attitudes towards their involvement in shared decision-making and their medical decisions [55]. The involvement of caregivers in shared medical decision-making could have a positive influence on patient medical decisions, reducing patient decision regret and better care for the patient [41,56].

From our results, it emerged that patients and caregivers experienced the cancer diagnosis as a shared shocking experience, with a sense of unity, that also negatively impacted both patients and caregivers on the psychological side [58,59,60,61,62,63,64,66,67]. During the cancer trajectory, patient–caregiver dyads experienced different phases: soon after the diagnosis they frequently retreated from normal life; then, they started to accept this new situation, tried to deal with it, and carried on as normal [57,58,60,62,64,65,66]. Although family caregivers provided both emotional and instrumental support, they also had to cope with their emotional needs regarding the diagnosis to be strong for the patients [57,59,63,64,65,66,67]. As a result, patients sometimes reported better scores in terms of psychological distress (e.g., anxiety and depression) than their caregiver [53]. Furthermore, there was an interdependence between the psychological aspects of patients and caregivers that did not influence the role that patients and caregivers wanted to play in the shared medical decision-making process [53]. Specifically, the cancer diagnosis and treatments overwhelmed patients and caregivers [5,16,17,18,57,63,64]. In particular, breast and prostate cancer patients were worried about sexuality and their intimacy, as already reported in the literature [4,5,7,8,9,19,60,61,63].

Finally, our results showed that along the cancer trajectory, patients and caregivers could disagree about whom to tell about cancer or about seeking a second opinion or because patients perceived the support provided by caregivers was inappropriate, causing tension or conflict [59,60,66].

### 4.1. Limitations

Before concluding, it is important to point out some limitations of the reviewed studies. Of the 22 included in this review, 11 were conducted in North America, 7 in Europe, 3 in Asia [55,56,68], and only 1 in Oceania [66]. Since most of the studies (18/2) included in this review were conducted in North America and Europe, the result may be limited in generalizability. Of the 5 longitudinal studies, with observation times ranging from 4 months to 18 months from baseline, none of the studies explored the entire trajectory of a family member’s caregiving experience as a dyad from cancer diagnosis to end of treatment (survivorship). Moreover, cross-sectional studies had different time points. Furthermore, it is difficult to draw final conclusions on the dyads’ psychological trends during the cancer-care pathway since the included studies used different time points of evaluation. Another limitation is that there was no balance between studies on prostate cancer and studies on breast cancer, so it was difficult to give a final conclusion. Finally, studies comparing the different roles and interdependence of the dyads among different cancers (lung, blood, and so on) might help in tailoring interventions according to the needs linked to the specific cancer diagnosis.

### 4.2. Recommendations for Future Research

The findings of the review indicate that caregiver involvement in cancer patients’ shared decision-making is very important because caregivers provide emotional and instrumental support to patients [57,63,64,65,66]. Moreover, patients and caregivers walk the path of treatment together and both feel negative psychological aspects that are interdependent [53,60,62,63,64]. Furthermore, cancer patients prefer to make medical decisions after consulting or sharing the decision made with their caregivers [41,53,54,55]. Considering this evidence, more research is needed to examine the interdependence among cancer dyads regarding psychological aspects, their role in the decision-making, and how they change over time according to different phases of care to facilitate the advancement of such a framework. Furthermore, given the recent COVID-19 pandemic and its countless restrictions, it could be interesting to evaluate the impact that the limitations imposed on joint visits had on the shared decision-making and on patients’ psychological well-being [69,70].

## 5. Conclusions

These findings highlight the importance of using a perspective that focuses on the relationship between a patient and caregivers when they receive a cancer diagnosis and must make a treatment decision. Targeting caregiver–patient dyads, rather than individuals, is important since a supported relationship could have a protective effect related to psychological distress, QOL, and relationship satisfaction. Moreover, dyads may benefit from interventions that focus on the needs of both the patient and caregiver.

## Figures and Tables

**Table 1:** Eligibility Criteria (with exclusion criteria).

	Included	Excluded
Topic	Caregiver involvement in breast and prostate cancer patients’ decision-making along their cancer care path.	
Research domain	Application to the specific field of clinical psychology (broadly understood).	
Population	Competent breast or prostate cancer patients and their caregivers (e.g., parents, partners/spouses, sons, other loved ones, or close friends).	Breast or prostate cancer patients and/or caregivers that are not competent due to cognitive impairment or coma. Patients with other cancers than breast and prostate.
Language	Publications in English, Italian, or Spanish.	Publications in other languages than English, Italian, and Spanish.
Types (research methodology)	Publications are one of the following types of literature: (a) qualitative literature (e.g., in-depth interview, semi-structured interview, focus group); (b) quantitative literature (e.g., survey, standardized questionnaire); (c) mixed methods literature.	Editorials, conferences, commentaries, book chapters, guidelines, and literature reviews

**Table 2:** Quantitative reports.

Authors, Year, Country	Data Collection Technique	Samples Time Points	Measurement Time-Point and Treatment	Significant Findings
Davison et al. 2003 Canada [53]	Control Preferences Scale (CPS) State Anxiety Inventory (STAI–Y Form) The Center for Epidemiologic Studies Depression Scale (CES-D).	148 participants: 74 PCp (μ_age_ = 62.2; 41–79) 74 Partners (μ_age_ = 58.1; 29–76)	At the time of diagnosis and 4 months later Treatment = 75% radical prostatectomy	**Shared decision-making** Most patients wanted to share the decision with their partner or to decide after consulting them and assumed a more active role in TDM than originally intended Almost all the partners preferred to play either a collaborative or passive role in the TDM and assumed a more passive role in the TDM than originally preferred. **Psychological impact** Patient and caregiver levels of psychological distress did not influence the role that they assumed vs. the roles they originally had preferred in TDM.
Davison et al. 2002 Canada [54]	Control Preferences Scale (CPS) regarding their physician and their partner Information Survey Questionnaire.	160 participants 80 PCp (μ_age_ = 61.3; 41–79) 80 caregivers (Partners) (μ_age_ = 57.2; 29–76)	After their initial treatment consultation Treatment = Not yet decided	**Shared decision-making** Most patients preferred to play either an active or a collaborative role in TDM. Partners preferred to play a collaborative role or passive role in TDM.
Gilbar and Gilbar 2009 Israel [55]	doctor–patient/spouse relationships decision making regarding medical treatment	114 participants *n* = 57 BCp (μ_age_ = 53; 34–69) *n* = 57 (husband) (μ_age_ = 54.42; 37–75)	3 to 12 months after diagnosis Treatment = under medical treatment	**Shared decision-making** Almost all the patients preferred to decide by themselves, but their decision should be in accord with that of their husband’s. Most of the patients thought that their husbands should participate in the TDM. Significant high correlation between patients and their husbands regarding participants in the decision-making, and who the important parties are in the decision-making process.
Kuo et al. 2019 China [56]	The Involvement in the Breast Reconstruction Decision-Making Process Scale The Body Image Scale The Decision Regret Scale	210 participants *n* = 105 BCp (μ_age_ = 48.3; 32–64) *n* = 105 (Partner) (μ_age_ = 50.59; 32–74)	16 months since breast reconstruction Treatment = Mastectomy	**Shared decision-making** Patients’ and partners’ decision involvements were significantly correlated. The dyadic decision involvement was significantly inversely related to decision regret
Veenstra et al. 2019 USA [41]	Questionnaire on decision support persons concerning 3 domains of engagement in decision making.	2406 participants *n* = 1203 BCp (μ_age_ = /) *n* = 1203 (43% Husband/Partner, 23% daughter. 23% other family members, 10% friends or other non-family members) (μ_age_ = /)	7 months after their diagnosis Treatment =31% CT, 50% RT, 62% lumpectomy, 38% mastectomy	**Shared decision-making** Partners were significantly more engaged than other types of decision support persons. Having a highly informed decision support person was associated with higher odds of greater patient-reported subjective decision quality Having a highly aware decision support person was associated with higher odds of a more deliberative decision.

Abbreviations: BCp = breast cancer patients; CT = chemotherapy; PCp = prostate cancer patients; RT = radiation therapy; TDM = treatment decision-making.

**Table 3:** Qualitative reports.

Authors, Year, Country	Data Collection Technique	Sample Time Points	Measurement Time-Point and Treatment	Significant Findings
Boehmer and Babayanc, 2005 USA [57]	Face to face or telephone interview	39 participants: 21 PCp (μ_age_ = 59.57; 37–70) 18 Caregiver = 14 F (12 wife, 1 partner, 1 daughter, 1 relative, 3 friends) (μ_age_ = 55.28; 39–70)	Patients had decided on a treatment modality, but had not yet started Treatment = 66.7% Surgery, 19% Brachytherapy, 14.29% RT	**Shared decision-making** Men received from their partners emotional and informational support (emerged as being significant during the pre-treatment phase) and involved them in medical appointments **Psychological impact** Spousal support depended on the diagnosed partner’s willingness to accept emotional and/or informational support.
Docherty et al., 2007 UK [58]	Focus group	12 participants 9 PCp (μ_age_ = 71; 54–79) 3 Partners (μ_age_ = 71; 54–79)	Time since treatment ranged from 6 months to 5 years Treatment = RT, orchidectomy, and HT	**Shared decision-making** Patients made their decision using their spouse’s support (often wife contacted the staff to gain information). Couples appreciated being involved in decisions and faced it as a team
Fasse et al., 2007 France [59]	Semi-directive interviews	18 participants *n* = 9 BCp (μ_age_ = 54; 33–66) *n* = 9 Partners (μ_age_ = 59; 40–76)	The time from diagnosis varied between 2 and 8 years Treatment = mastectomy	**Shared decision-making** The DM on BR did not entirely depend on patients’ and caregivers’ wishes and expectations. Some women and a few partners argued that the TDM was something decided by someone else, not necessarily causing distress or upset for the participants.
Gray et al., 1999 Canada [60]	Interview	68 participants *n*= 34 PCp (μ_age_ = 60.6; 50–68) *n* = 34 Partners (μ_age_ = 57; 42–72)	Waiting for surgery /	**Shared decision-making** Some couples took time to make decisions, investigating together different conventional and unconventional options along the way. **Psychological impact** The PC diagnosis came as a shock for all the couples, experiencing it as a shared one. It was frequently accompanied with a partial retreat from normal life. The absence of any sense of reconnection between the partners seemed to create dissonance in the relationship. Sometimes couples felt it was important to discuss what scared them and how they felt, and then to reassure each other
Lamore et al., 2020 France [61]	Semi-structured interview	18 participants *n* = 9 BCp (μ_age_ = 54; 39–66) *n* = 9 Partner (μ_age_ = 59; 40–76)	After mastectomy Treatment = Surgery	**Shared decision-making** The DM for BR seems linked to the family history of cancer, and temporality is important. Couples needed to talk together, or with other people about the mastectomy and the BR. **Psychological impact** Participants were concerned about treatments and their effects on their relationship.
Le et al., 2016 USA [62]	Semi-structured telephone interviews	30 participants *n* = 15 PCp (μ_age_ = 61.5; 49–72) *n* = 15 Wife (μ_age_ = 59.3; 45–71)	Within 6–18 months of the decision Treatment = 10 AS, 3 RT, 2 Surgery	**Shared decision-making** Most of the couples perceived the DM to be collaborative. PCp shared responsibility and partners reported that patients made the decision after considering their point of view. Men who chose AS were more likely to make the decision on their own than those who chose active treatment. **Psychological impact** Couples reported high dyadic adjustment, finding their marital interactions satisfying and their relationships cohesive All couples described similar sequences of a highly emotional initial reaction and desire to be rid of the cancer, information seeking, and decision making.
Loaring et al., 2015 UK [63]	Interviews	8 participants *n* = 4 BCp (μ_age_ = /; 37–55) *n* = 4 Partners (μ_age_ = /; 37–55)	At least 6 months post treatment Treatment = Mastectomy	**Shared decision-making** Patients were seen by men as decision-makers. Partners wanted to be part of TDM, but they put their wives’ needs or preferences first and supported whatever decision they made. Husbands were active and involved in TDM. **Psychological impact** There was a sense of “being together” and having a shared understanding of cancer. The diagnosis and the decisions regarding treatment could be overwhelming. Patients were concerned about their bodies and they did not believe that their husbands could find them attractive, even if partners found their wives as attractive as they did before breast cancer.
Maliski et al., 2002 USA [64]	Interview	40 participants *n* = 20 PCp (μ_age_ = 58.9; 51–71) *n* = 20 Wife (μ_age_ = 54.3; 28–70)	Between 3- and 11-months post prostatectomy Treatment = Prostatectomy	**Shared decision-making** Patients made their decision searching information on outcomes and complications of each treatment. The final choice of treatment was made by the PCp, even if they discussed treatment with their wives. **Psychological impact** The wives’ goal was to support their husbands’ decision, but they were concerned about their ability to do so. The diagnosis of PC represented a loss of control. Cancer was a shock, trauma, disbelief, and couples were scared.
Nelson et a., 2019 USA [65]	Semi-structured interviews	36 participants *n* = 18 PCp (μ_age_ = 11.1% 50–59, 50% 60–69, 37.5% 70–70; /) *n* = 18 Partners (μ_age_ = 44.4% 50–59, 44.4% 60–69, 11.1% 70–70; /)	At 3 time-points following diagnosis Treatment = Active surveillance 5 Radical prostatectomy 6 Hormone/radiotherapy 7	**Shared decision-making** Men on AS preferred not to discuss their cancer once treatment decisions had been made. **Psychological impact** Throughout the illness trajectory, partners provided emotional, instrumental, and appraisal support and they reported being happy to provide support. Partners helped patients to deal with treatment-related side effects (sexual dysfunction). Men receiving treatment spoke favorably about the support they had received from their partner. Some partners mentioned a perception that much of the support on offer was superficial and found it difficult to accept support from their wider network.
O’Callaghan et al., 2014 Australia [66]	Interview	35 participants *n* = 21 PCp (μ_age_ 4.76% ≤50, 28.57% 51–60, 52.38% 61–70, 14.29 >71; /) *n* = 14 Partners (μ_age_ = 28.57% ≤50, 14.29% 51–60, 50% 61–70, 7.14% >71; /)	/ Treatment = 11 still on AS, 7 RP after ≥3 months on AS, 1 EBRT after ≥3 months on AS, 1 BT after ≥3 months on AS, 1 RP immediately after diagnosis	**Shared decision-making** Men’s TDM were informed by perspectives from medical staff and caregivers and affected by their emotional reactions, cancer-related memories, and lifestyle factors. Partners supported men’s final decisions, who usually felt the support provided by caregivers. **Psychological impact** Men and partners’ strategies for coping: positive self-talk, living as normally as possible, distraction, thinking of PC survivors, rationalizing that one could die of something else, hope for new PC treatments, denial, educating others about PC, acquiring information, continuing a healthy lifestyle, seeking reassurance, and humor.
Rim et al., 2011 USA [67]	Focus Group	433 participants *n* = 240 PCp (μ_age_ = less than 60–more than 70) *n* = 193 Caregivers (93% Wife/Partner, 5% Daughter/Son, 3% Other/Unknown) (μ_age_ = less than 60- more than 70)	At diagnosis /	**Shared decision-making** Some patients found partners’ preference for a particular treatment “very important” Family members reported discussing treatment options with patients “very often”. Nearly all family members “strongly agreed” their role was to listen and provide emotional support, to help obtain information about cancer and treatment options, to arrange meetings with physicians and other health professionals, and to help patients make a treatment decision.

Abbreviations: BCp = breast cancer patients; BR = breast reconstruction; CT = chemotherapy; FG = focus group; HT = hormone-therapy; PCp = prostate cancer patients; PC = prostate cancer; RT = radiation therapy.

**Table 4:** Mixed methods report.

Authors, Year, Country	Data Collection Technique	Sample Time Points	Measurement Time-Point and Treatment	Significant Findings
Al-Bahri et al., 2019 Oman [68]	CanCORS questionnaires	158 participants: 79 BCp (μ_age_ = 45; 26–75) 79 Caregiver (39.2 % spouses, 17.7% siblings, 34.2% parents, 8.9% others) (μ_age_ = 36; 18–65)	Two years after treatment Treatment = 59.5% surgery; 40.5% other treatments	**Shared decision-making** More family members than the patients reported equally-shared the TDM and high levels of family control in the TDM. Most of the BC patients shared the TDM with more than one family member. Most patients reported that their families usually come together to discuss and finalize the TDM, which occurred when there was full agreement between their family members.

## Data Availability

The data presented in this study are available on request from the corresponding author.

## Appendix A

**Table A1:** Search Strategy.

Search Strategy for Scientific Literature				
Search Engine:	Search String:	Hits	Relevant ^a^	Included ^b^
Pubmed	(“role” OR “experience” OR “involvement”) AND (“decision making” OR “choices” OR “decisions”) AND (“caregiver” OR “partner” OR “family member” OR “spouse” OR “carer” OR “relative”) AND (“cancer” OR “neoplasm”) AND (“breast” OR “prostate”) AND (“adult” OR “middle age” OR “very elderly” OR “young adult”) AND (“english” OR “italian” OR “Spanish”)	767	66	15
Embase	(‘role’/exp OR role OR ‘experience’/exp OR experience OR ‘involvement’/exp OR involvement) AND ((‘decision’/exp OR decision) AND making OR ‘choice’/exp OR choice OR ‘decision’/exp OR decision) AND ((‘caregiver’/exp OR caregiver OR ‘partner’/exp OR partner OR ‘family’/exp OR family) AND member OR ‘spouse’/exp OR spouse OR ‘carer’/exp OR carer OR ‘relative’/exp OR relative) AND (‘cancer’/exp OR cancer OR ‘neoplasm’/exp OR neoplasm) AND (‘breast’/exp OR breast OR ‘prostate’/exp OR prostate)) AND ([adult]/lim OR [aged]/lim OR [middle aged]/lim OR [very elderly]/lim OR [young adult]/lim) AND ([english]/lim OR [italian]/lim OR [spanish]/lim)) AND [embase]/lim	251	76	2
	1. role.mp. [mp = title, abstract, original title, name of substance word, subject heading word, floating sub-heading word, keyword heading word, organism supplementary concept word, protocol supplementary concept word, rare disease supplementary concept word, unique identifier, synonyms] (103414) 2 experience.mp. [mp = title, abstract, original title, name of substance word, subject heading word, floating sub-heading word, keyword heading word, organism supplementary concept word, protocol supplementary concept word, rare disease supplementary concept word, unique identifier, synonyms] (26523) 3 involvement.mp. [mp = title, abstract, original title, name of substance word, subject heading word, floating sub-heading word, keyword heading word, organism supplementary concept word, protocol supplementary concept word, rare disease supplementary concept word, unique identifier, synonyms] (14510) 20 4 and 8 and 15 and 16 and 19 (8)			
PsycINFO	1 ((role or experience or involvement) and (decision making or choices or decisions) and (caregiver or partner or family member or spouse or carer or relative) and (cancer or neoplasm) and (breast or prostate)).mp. [mp = title, abstract, heading word, Search Strategy of contents, key concepts, original title, tests and measures, mesh] (65) 2 limit 1 to (adulthood <18+ years> and (“300 adulthood <age 18 yrs and older > “ or 320 young adulthood <age 18 to 29 yrs> or 360 middle age <age 40 to 64 yrs > ) and (english or italian or spanish)) (48)	48	7	0
Subtotal		1160	150	17
Duplicates		239		
Excluded due to missing data		3		
Total		918	150	17

^a^ Relevant: number of relevant articles based on title, abstract, and keywords. ^b^ Included: number of included articles based on full article.
